# Hepatic expression of multidrug resistance protein 2 in biliary atresia

**DOI:** 10.1186/1476-5926-10-6

**Published:** 2011-08-03

**Authors:** Keita Terui, Takeshi Saito, Tomoro Hishiki, Yoshiharu Sato, Tetsuya Mitsunaga, Hideo Yoshida

**Affiliations:** 1 Department of Pediatric Surgery, Graduate School of Medicine, Chiba University, Chiba, Japan

## Abstract

**Background:**

Biliary atresia (BA) is an idiopathic inflammatory obliterative cholangiopathy of neonates, leading to progressive biliary cirrhosis. Hepatoportoenterostomy (Kasai procedure) can cure jaundice in 30% to 80% of patients. Postoperative clearance of jaundice is one of the most important factors influencing long-term outcomes of BA patients. Multidrug resistance protein 2 (MRP2) is one of the canalicular export pumps located in hepatocytes; it exports organic anions and their conjugates (e.g., bilirubin) into bile canaliculus. Although MRP2 is an essential transporter for the excretion of bilirubin, its role in the clinical course of BA patients is unclear. The present study investigated the relationship between hepatic MRP2 expression and clinical course in BA patients, with particular emphasis in curing jaundice after hepatoportoenterostomy.

**Results:**

No significant differences in hepatic MRP2 expression level were observed between BA and controls groups. There was no correlation between MRP2 expression and age at time of surgery in BA and control groups. In BA patients, MRP2 expression level in the jaundice and jaundice-free group did not differ significantly (2.0 × 10^-4 ^vs 3.1 × 10^-4^, p = 0.094). Although the serum level of total bilirubin just before surgery did not correlate with MRP2 expression level (rs = 0.031, p = 0.914), the serum level of total bilirubin measured at 2 weeks (rs = -0.569, p = 0.034) and 4 weeks after surgery (rs = -0.620, p = 0.018) were significantly correlated with MRP2 expression level. Furthermore, MRP2 expression level was inversely correlated with ratio of change in serum total bilirubin level over 4 weeks (rs = -0.676, p = 0.008), which represents the serum bilirubin level measured at 4 weeks after surgery divided by value just before surgery. There was no correlation between expression level of MRP2 and nuclear receptors, such as retinoid × receptor α, farnesoid × receptor, pregnane × receptor, or constitutive androstane receptor.

**Conclusions:**

Hepatic MRP2 expression level was associated with postoperative clearance of jaundice in BA patients, at least within 1 month after hepatoportoenterostomy. This finding suggests that not only morphological appearance of the liver tissue but also the biological status of hepatocytes is important for BA pathophysiology.

## Background

Biliary atresia (BA) is an idiopathic inflammatory obliterative cholangiopathy of neonates, leading to progressive biliary cirrhosis [1]. If performed within the first 3 months of life, hepatoportoenterostomy (Kasai procedure) can cure jaundice in 30% to 80% of patients [2-4]. Postoperative clearance of jaundice is one of the most important factors influencing long-term outcomes of BA patients. A multicenter study of 104 BA patients revealed that patients with total bilirubin level < 2 mg/dl at 3 months after hepatoportoenterostomy had significantly better prognoses than those with higher bilirubin levels (native liver survival, 84% vs 16%) [5].

Studies have aimed to predict the outcome of hepatoportoenterostomy for BA based on several variables, such as age at surgery, microscopic analysis of the resected specimen, and bile biochemistry [6]. Although age at surgery is an influential variable [1], evidence from a large series revealed that children with isolated BA showed no statistical difference by age cohort for clearance of jaundice or for native liver survival [1,7]. Furthermore, actual time of biliary occlusive onset might vary between prenatal and postnatal cases. Histopathological findings in the transected remnant, and in particular the size of the biliary ductules, has been thought to be a predictor of restoration of bile flow [6,8-10]. However, because of difficulties with consistency of histological interpretation, this result can be difficult to estimate in individual cases [1].

Recent studies of the molecular mechanisms of bile physiology have provided a better understanding of the pathophysiology of various cholestatic liver diseases. Different transporters are involved in bile secretion, and hepatobiliary transport systems are responsible for hepatic uptake and excretion of various endo- and xenobiotics including bile salts, bilirubin, cholesterol, phospholipids, hormones, and drugs. Multidrug resistance protein 2 (MRP2), which belongs to the ATP-binding cassette transporter superfamily (sub-family C, member 2: ABCC2) is one of the canalicular export pumps located in hepatocytes; it exports organic anions and their conjugates into bile canaliculus [11,12]. Clinically, dysfunction of MRP2 is known to result in hyperbilirubinemia. Hereditary deficiency of MRP2, known as Dubin-Johnson syndrome, causes hyperbilirubinemia [13]. The risk of intrahepatic cholestasis of pregnancy is associated with single nucleotide polymorphisms of MRP2 [14]. Hepatic expression of MRP2 in patients with primary sclerosing cholangitis was decreased compared with non-cholestatic controls [15]. Furthermore, a study in adult patients with biliary cancer has shown that impaired expression of hepatic MRP2 is associated with posthepatectomy hyperbilirubinemia [16]. Thus, MRP2 is one of the most essential transporters for excretion of bilirubin. However, the role of MRP2 in the clinical course of BA patients has not been elucidated.

The present study was designed to investigate the relationship between hepatic MRP2 expression and the clinical course of BA patients. In particular, the role of MRP2 in clearance of jaundice after hepatoportoenterostomy was studied. Furthermore, we assessed the association between expression levels of MRP2 and nuclear transporters, which are involved in the transcriptional regulation of MRP2.

## Results

### Clinical background

**Figure 1:**
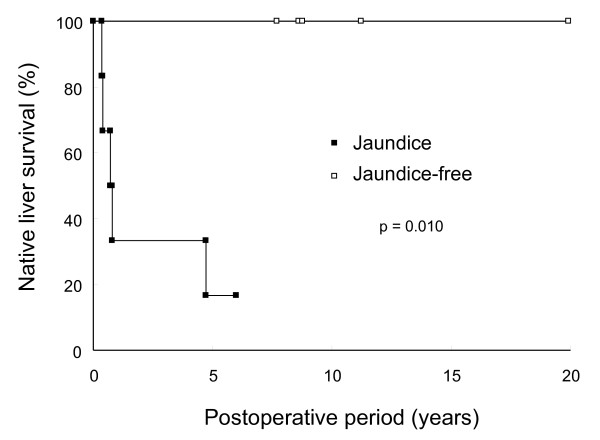
**Native liver survival of jaundice and jaundice-free group in BA patients**. Native liver survival differ significantly between the jaundice and jaundice-free groups (p = 0.010).

The clinical parameters of the three groups of patients (BA with jaundice, BA without jaundice, and controls) are shown in Table 1. Age at sampling of the jaundice and jaundice-free group were (mean ± SEM) 70.6 ± 8.7 and 76.8 ± 11.4 days respectively (p = 0.619). Five of 11 BA patients underwent liver transplantation during a follow-up of 8.5 ± 1.2 years. There was no difference of age at sampling between those who survived without transplantation and those who survived with transplantation (p = 0.366). Native liver survival differ significantly between the jaundice and jaundice-free groups (p = 0.010) (Figure 1).

**Table 1:** Clinical parameters in the jaundice, jaundice-free, and control groups

	Jaundice	Jaundice-free	Control
	n = 9	n = 5	n = 13
Age at sampling (days)			
Serum level of total bilirubin (mg/dl)	70.6 ± 8.7	76.8 ± 11.4	852.1 ± 101.3
Before sampling	10.7 ± 1.3	7.7 ± 2.5	0.7 ± 0.1
1 month after sampling	6.4 ± 1.0	3.1 ± 1.1	0.5 ± 0.0
3 months after sampling	4.6 ± 1.5*	0.8 ± 0.2	0.5 ± 0.1

*Except for 3 samples, from patients that underwent hepatoportoenterostomy followed by a secondary surgical procedure within 3 months.

### Hepatic expression of MRP2 and nuclear receptors

**Figure 2:**
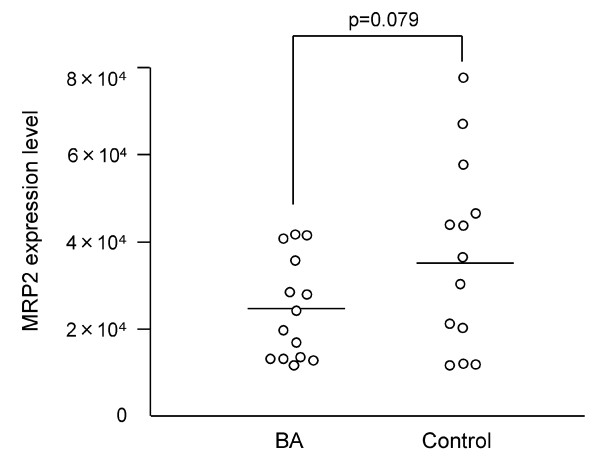
**Hepatic MRP2 expression level of BA patients and controls**. MRP2 expression level did not differ significantly between the BA and control groups (2.4 × 10^-4 ^± 3.1 × 10^-5 ^vs 3.7 × 10^-4 ^± 6.0 × 10^-5^, p = 0.079). Crossbars indicate median values.

**Figure 3:**
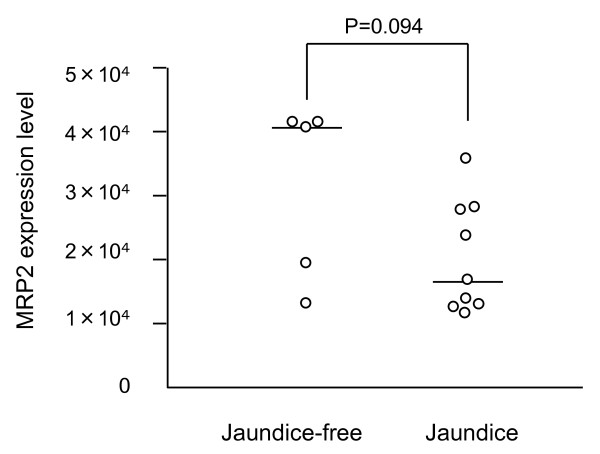
**Hepatic MRP2 expression level of jaundice and jaundice-free group in BA patients**. MRP2 expression level did not differ significantly between the jaundice and jaundice-free groups (2.0 × 10^-4 ^± 2.9 × 10^-5 ^vs 3.1 × 10^-4 ^± 6.2 × 10^-5^, p = 0.094). Crossbars indicate median values.

No significant difference in MRP2 expression level was observed between BA and control patients (2.4 × 10^-4 ^± 3.1 × 10^-5 ^vs 3.7 × 10^-4 ^± 6.0 × 10^-5^, p = 0.079) (Figure 2). There was no correlation between MRP2 expression and age at time of surgery in the BA (rs = 0.503, p = 0.067) or control group (rs = 0.514, p = 0.073). MRP2 expression levels in the jaundice and jaundice-free group were 2.0 × 10^-4 ^± 2.9 × 10^-5 ^and 3.1 × 10^-4 ^± 6.2 × 10^-5 ^respectively (p = 0.094) (Figure 3). There was no difference of MRP2 expression between those who survived without transplantation and those who survived with transplantation (p = 0.078). The levels of GAPDH expression were not different between BA patients and controls, between jaundice and jaundice-free group in BA patients, and between those who survived without transplantation and those who survived with transplantation or died.

**Figure 4:**
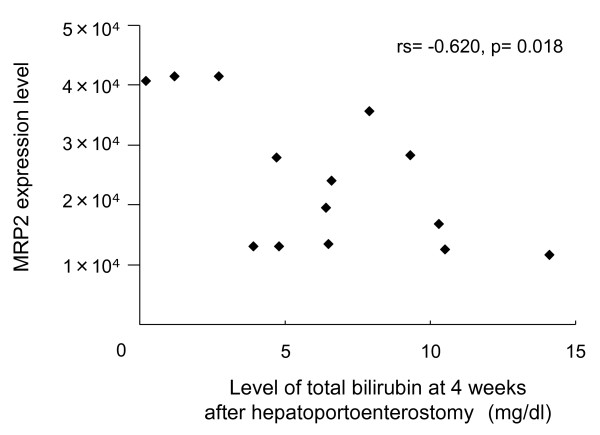
**Association between hepatic MRP2 expression level and level of total bilirubin at 4 weeks after surgery**. MRP2 expression levels correlated with serum levels of total bilirubin measured at 4 weeks after surgery (rs = -0.620, p = 0.018).

Next, the association between MRP2 expression and the serum level of total bilirubin in the perioperative period was assessed. The serum level of total bilirubin just before surgery did not correlate with MRP2 expression level (rs = 0.031, p = 0.914). The serum level of direct bilirubin just before surgery also had no correlation (rs = -0.016, p = 0.956). The serum level of total bilirubin measured at 2 weeks (rs = -0.569, p = 0.034) and 4 weeks after surgery (rs = -0.620, p = 0.018) correlated with MRP2 expression levels (Figure 4). The serum level of direct bilirubin measured at 4 weeks after surgery (rs = -0.577, p = 0.031) also correlated with MRP2 expression levels, but that measured at 2 weeks after the surgery did not (rs = -0.477, p = 0.085). Furthermore, MRP2 expression levels were also inversely correlated with ratio of change in the serum level of total bilirubin during the 4 weeks after surgery (rs = -0.676, p = 0.008), which represent the serum level of bilirubin measured at 4 weeks after surgery divided by value just before surgery. The ratio of change in the serum level of direct bilirubin during 2 weeks (rs = -0.543, p = 0.045) and 4 weeks (rs = -0.586, p = 0.028) also correlated with MRP2 expression levels, although values of total bilirubin during 2 weeks did not.

**Figure 5:**
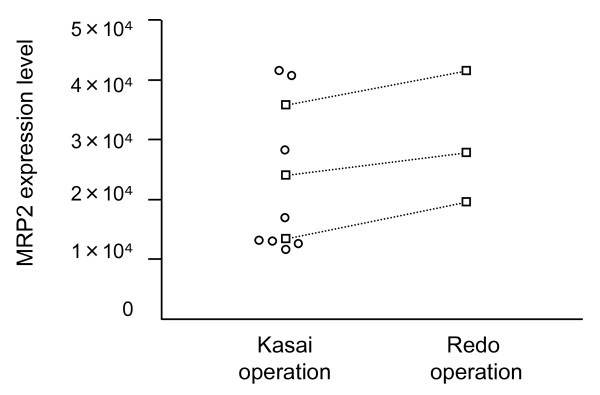
**Hepatic MRP2 expression level of BA patients at the time of hepatoportoenterostomy and secondary surgical procedure**. Squares indicate patients who underwent both hepatoportoenterostomy and a secondary surgical procedure. In these 3 cases, MRP2 expression level at the secondary surgical procedure increased compared with levels seen at the initial hepatoportoenterostomy.

The data in Figure 5 shows MRP2 expression level of BA at primary hepatoportoenterostomy and at a secondary surgical procedure, respectively. Although statistical analysis could not be applied because of the small number of patients, in all 3 cases that underwent 2 surgical procedures, MRP2 expression level at the secondary procedure increased compared with levels at the first hepatoportoenterostomy.

There was no correlation between expression level of MRP2 and any nuclear receptor: RXRα (rs = -0.164, p > 0.05), FXR (rs = 0.373, p > 0.05), PXR (rs = 0.409, p > 0.05) and CAR (rs = 0.0257, p = 0.940).

## Discussion

MRP2 is one of the canalicular export pumps located in hepatocytes; it exports organic anions and their conjugates (e.g., bilirubin) into bile canaliculus [11,12]. The effect of MRP2 is regulated at transcriptional and posttranscriptional levels in response both to many endogenous and xenobiotic substances and to abnormal states, such as biliary obstruction and inflammation [17,18]. Biliary obstruction initiates marked changes in transporter expression, which is reasonable for hepatic protection [19]. Basolateral transporters for bile acid uptake are downregulated to prevent further uptake, and the canalicular export pump, MRP2, is also downregulated. Alternatively, basolateral transport systems such as MRP3 and 4 are compensatively upregulated to prevent accumulation of potentially toxic substrates in hepatocytes [20]. Secretion of interleukin-1β (IL-1β) induced by obstructive cholestasis is responsible for reduced transcription of MRP2 via decreased binding RXRα to the MRP2 promoter [21,22]. Meanwhile, inflammatory status induced by proinflammatory cytokines, including tumor necrosis factor α, IL-1β, and IL-6, also results in reduced bile flow via changing gene expression of transporters [23,24]. MRP2 expression is downregulated drastically in cytokinemia induced by endotoxin administration [25-27]. In addition, MRP2 expression level in the BA liver was reported to be downregulated compared with age-matched controls that had non-cholestatic liver diseases [28]. In the present study, we found no significant difference of MRP2 expression between BA and control. Our result might be influenced by selection of controls; the average age of controls was much older than that of BA patients. Considering the age dependency of canalicular transporters, including MRP2 especially in small infants [17], the difference of ages might have affected the results. Furthermore, the controls include liver samples from choledochal cyst, potentially an obstructive cholestatic disease, although the cases of choledochal cyst that had jaundice at the sampling were excluded in the study.

The pathophysiology of BA is characterized as inflammatory obliterative cholangiopathy [1]. Immunohistochemical studies have revealed that activated T cells infiltrate the periductal area with expression of various intracellular adhesion molecules [29,30]. In the present study, we showed that a higher hepatic MRP2 expression level at the time of surgery resulted in faster clearance of jaundice with lower serum levels of bilirubin within a month of surgery. It is still unclear what caused difference of MRP2 expression in the BA liver. Considering the molecular mechanisms of bile physiology, prolonged biliary obstruction and/or advanced inflammatory status might have effect on it, but further studies are still necessary. Meanwhile, the level of MRP2 expression was not involved in long-term prognosis. The discrepancy between clearance of jaundice and prognosis could be partially explained by a small number of cases. And, prognosis of native liver of BA is also influenced by other factors, such as liver fibrosis, portal hypertension, and cholangitis. Regardless, MRP2 is an important molecule in understanding the biological status of the BA livers, and also important clinically because sufficient clearance of jaundice is necessary for a positive long-term prognosis.

Transcriptional regulation may result from changes in the intracellular concentrations of bile acids and a number of lipophilic compounds that are ligands for nuclear receptors. The key nuclear receptors influencing MRP2 expression are RXRα, FXR, PXR, and CAR [31,32]. We showed no correlation between expression level of MRP2 and any nuclear receptor. This led us to think that the difference of MRP2 expression level in BA patients did not result from transcriptional changes of nuclear receptors. Meanwhile, posttranscriptional effects of nuclear receptors activated by various agonists have been elucidated in various animal models. Controlling the effect of transporters via nuclear receptors may be an approach to developing new drugs for cholestatic liver disease [33].

In all BA patients who underwent a secondary surgical procedure, MRP2 expression level increased after the first operation, although jaundice worsened. All 3 cases received ursodeoxycholic acid (UDCA) (20 mg/kg/day) after hepatoportoenterostomy. Although the mechanism of the anti-cholestatic effects of UDCA are not clearly understood, UDCA-induced transcriptional upregulation of MRP2 and insertion of transporter molecules including MRP2 into the canalicular membrane of hepatocytes have been reported [34]. UDCA might act to maintain MRP2 expression during cholestasis.

## Conclusions

Hepatic MRP2 expression level was associated with postoperative clearance of jaundice in BA patients within 1 month after hepatoportoenterostomy. This finding suggests that not only morphological appearance of the liver tissue but also the biological status of hepatocytes is important for BA pathophysiology. It remains unclear how MRP2 expression is regulated in the BA liver, and whether postoperative clearance of jaundice is directly associated with MRP2 expression. This retrospective preliminary report indicates that further study is necessary to elucidate the involvement of MRP2 in BA pathophysiology.

## Methods

### Patients and tissue specimens

Fourteen liver samples from 11 patients with BA treated in our institution from October 1998 to February 2005 were used. Diagnosis of BA was made based on surgical findings. The type of BA consisted of type 3 (n = 10) and type 1 (n = 1). There was no case with associated anomalies (e.g., splenic malformation, situs inversus). All surgeries were performed by 2 expert surgeons, and there were no critical complications in the perioperative period. Eleven samples were obtained during hepatoportoenterostomy, which was performed at a mean age of 65.5 days (range, 21 to 128 days). Of these 11 cases, 3 cases underwent a second hepatoportoenterostomy, and 3 samples were taken at that time (mean age at second surgery, 90 days; range, 64 to 114 days). The indication for the secondary procedures in our institution is postoperative jaundice which seems to be caused by fibrotic tissue at the hepatoportoenterostomy. There was no other indication, such as postoperative bleeding or anastomotic leakage.

Serum levels of bilirubin in patients with BA were reviewed, and BA samples were divided into two groups on the basis of postoperative results: jaundice group (n = 9) and jaundice-free group (n = 5). "Jaundice-free" was defined as serum levels of total bilirubin < 1.5 mg/dl within 3 months postoperatively. Three samples from the primary hepatoportoenterostomy followed by secondary surgical procedures were classified into the jaundice group. After the secondary hepatoportoenterostomy, two of three cases had serum levels of total bilirubin < 1.5 mg/dl within 3 months after surgery, and therefore, were classified into the jaundice-free group. The other one case was classified into the jaundice group. A sample of a case of type 1 BA (from primary hepatoportoenterostomy) was included in jaundice-free group.

Pediatric control samples were collected from 13 patients with liver diseases in the same way. They consisted of patients with choledochal cysts (n = 9) and hepatoblastoma (n = 4). The mean age of controls was 25.3 months (range, 2 to 54 months). Samples from choledochal cysts were obtained during excision of the cyst and hepatojejunostomy. Samples from hepatoblastoma included normal parts of the liver adjacent to tumorous lesions. None of the control patients were jaundiced at the time of sampling.

The study protocol was approved by the institutional ethics committee of Chiba University, and informed consent was obtained from the parents of all patients.

### Quantitative reverse transcription polymerase chain reaction

The liver samples were divided into two parts: one was frozen immediately stored at -80°C until RNA extraction, and the second was fixed in 10% buffered formaldehyde solution for pathologic estimation. Total RNA was extracted from the frozen liver using an Isogen reagent (Nippon Gene, Tokyo, Japan). First-strand cDNA synthesis was performed with reverse transcriptase, 5 mg of total RNA, and oligo (dT) primers. Quantitative reverse transcription polymerase chain reaction (qRT-PCR) was performed using the Universal ProbeLibrary Set and LightCycler 350S system (Roche, Mannheim, Germany). All cDNA samples were diluted 15-fold as a working template in qRT-PCR. Unique probe and gene-specific primer pair combinations for target genes were designed using Roche ProbeFinder Software Version 2.32. The primer sequences and probes were follows: MRP2, 5'-AGCATGCTTCCCATGATGA-3' and 5'-TCTAGCCGCTCTGTGGAAAC-3' (Probe#1); glyceraldehyde-3-phosphate dehydrogenase (GAPDH), 5'-AGCCACATCGCTCAGACA-3' and 5'-GCCCAATACGACCAAATCC-3' (Probe#60); retinoid × receptor α (RXRα), 5'-ACATGCAGATGGACAAGACG-3' and 5'-GAGAGCCCCTTGGAGTCAG-3' (Probe#27); farnesoid × receptor (FXR), 5'-TGACCTGTGAGGGGTGTAAAG-3' and 5'-CACTTGTACACAGCGTTTTTGG-3' (Probe#2); pregnane × receptor (PXR), 5'-ATGCAAGGGCTTTTTCAGG-3' and 5'-GGGTCTTCCGGGTGATCT-3' (Probe#30); constitutive androstane receptor (CAR), 5'-TAATGCGCTGACTTGTGAGG-3' and 5'-AGGTGGGACCAATGCTTTT-3' (Probe#2). Briefly, 1 × Probes Master, 200 nM of each primer, 100 nM Universal ProbeLibrary probe, and 2 μl diluted cDNA template were added to each reaction in a total volume of 20 μl. The protocol consisted of an initial denaturation step at 95°C for 10 min, followed by 40 cycles of amplification and quantification at 95°C for 15 s, 60°C for 10 s, and 72°C for 10 s, and was finally cooled at 40°C. The transcript amounts were estimated from the respective standard curves and normalized to the GAPDH transcript amount determined in corresponding samples. Reactions were run in duplicate.

### Statistical analysis

Results are presented as mean ± SEM. Differences of mean expression levels between groups were compared with the student t-test or Welch's t-test. Associations were assessed by Pearson's correlation coefficient test or Spearman's rank-correlation coefficient test, and expressed by the corresponding correlation coefficient (rs). Curves of native liver survival were calculated using Kaplan-Meier methodology and log rank test was used to compare survival rates. P values < 0.05 were considered significant.

## Competing interests

The authors declare that they have no competing interests.

## Authors' contributions

KT, TS and TH collected liver samples; YS and TM performed qRT-PCR; KT performed the statistical analysis and wrote the manuscript; and HY designed the study and reviewed the manuscript. All the authors have read and approved the final manuscript.

## References

[B1] Hartley JL Davenport M Kelly DA Biliary atresia Lancet 2009 374 1704 1713 10.1016/S0140-6736(09)60946-6 19914515

[B2] Schweizer P Treatment of extrahepatic biliary atresia: results and long-term prognosis after hepatic portoenterostomy Pediatr Surg International 1986 1 30 36

[B3] Ohi R Biliary atresia: a surgical perspective Clin Liver Dis 2000 4 779 804 10.1016/S1089-3261(05)70141-0 11232357

[B4] Sokol RJ Mack C Narkewicz MR Karrer FM Pathogenesis and outcome of biliary atresia: current concepts J Pediatr Gastroenterol Nutr 2003 37 4 21 10.1097/00005176-200307000-00003 12827000

[B5] Shneider BL Brown MB Haber B Whitington PF Schwarz K Squires R Bezerra J Shepherd R Rosenthal P Hoofnagle JH Sokol RJ Biliary Atresia Research Consortium A multicenter study of the outcome of biliary atresia in the United States, 1997 to 2000 J Pediatr 2006 148 467 474 10.1016/j.jpeds.2005.12.054 16647406

[B6] Davenport M Howard ER Macroscopic appearance at portoenterostomy-a prognostic variable in biliary atresia J Pediatr Surg 1996 31 1387 1390 10.1016/S0022-3468(96)90835-0 8906668

[B7] Davenport M Caponcelli E Livesey E Hadzic N Howard E Surgical outcome in biliary atresia: etiology affects the influence of age at surgery Ann Surg 2008 247 694 698 10.1097/SLA.0b013e3181638627 18362634

[B8] Gautier M Jehan P Odievre M Histologic study of biliary fibrous remnants in 48 cases of extrahepatic biliary atresia: correlation with postoperative bile flow restoration J Pediatr 1976 9 704 709 10.1016/s0022-3476(76)80787-1 978315

[B9] Hitch DC Shikes RH Lilly JR Determinants of survival after Kasai's operation for biliary atresia using actuarial analysis J Pediatr Surg 1979 14 310 314 10.1016/S0022-3468(79)80489-3 480093

[B10] Matsuo S Ikeda K Yakabe S Nakagawara A Iwashita A Histological study of the remnant of porta hepatis in patients with extrahepatic biliary atresia-Computed picture analysis of 30 cases Z Kinderchir 1984 39 46 49 6730701 10.1055/s-2008-1044168

[B11] Jansen PL Sturm E Genetic cholestasis, causes and consequences for hepatobiliary transport Liver Int 2003 23 315 322 10.1034/j.1478-3231.2003.00856.x 14708891

[B12] Trauner M Wagner M Fickert P Zollner G Molecular regulation of hepatobiliary transport systems: clinical implications for understanding and treating cholestasis J Clin Gastroenterol 2005 39 S111 S124 10.1097/01.mcg.0000155551.37266.26 15758646

[B13] Paulusma CC Kool M Bosma PJ Scheffer GL ter Borg F Scheper RJ Tytgat GN Borst P Baas F Oude Elferink RP A mutation in the human canalicular multispecific organic anion transporter gene causes the Dubin-Johnson syndrome Hepatology 1997 25 1539 1542 10.1002/hep.510250635 9185779

[B14] Sookoian S Castaño G Burgueño A Gianotti TF Pirola CJ Association of the multidrug-resistance-associated protein gene (ABCC2) variants with intrahepatic cholestasis of pregnancy J Hepatol 2008 48 125 132 10.1016/j.jhep.2007.08.015 17997497

[B15] Oswald M Kullak-Ublick GA Paumgartner G Beuers U Expression of hepatic transporters OATP-C and MRP2 in primary sclerosing cholangitis Liver 2001 21 247 253 10.1034/j.1600-0676.2001.021004247.x 11454187

[B16] Yamada T Arai T Nagino M Oda K Shoda J Suzuki H Sugiyama Y Nimura Y Impaired expression of hepatic multidrug resistance protein 2 is associated with posthepatectomy hyperbilirubinemia in patients with biliary cancer Langenbecks Arch Surg 2005 390 421 429 10.1007/s00423-005-0564-5 15965653

[B17] Fardel O Jigorel E Le Vee M Payen L Physiological, pharmacological and clinical features of the multidrug resistance protein 2 Biomed Pharmacother 2005 59 104 114 10.1016/j.biopha.2005.01.005 15795103

[B18] Nies AT Keppler D The apical conjugate efflux pump ABCC2 (MRP2) Pflugers Arch 2007 453 643 659 10.1007/s00424-006-0109-y 16847695

[B19] Wagner M Zollner G Trauner M New molecular insights into the mechanisms of cholestasis J Hepatol 2009 51 565 580 10.1016/j.jhep.2009.05.012 19595470

[B20] Geier A Wagner M Dietrich CG Trauner M Principles of hepatic organic anion transporter regulation during cholestasis, inflammation and liver regeneration Biochim Biophys Acta 2007 1773 283 308 10.1016/j.bbamcr.2006.04.014 17291602

[B21] Denson LA Auld KL Schiek DS McClure MH Mangelsdorf DJ Karpen SJ Interleukin-1beta suppresses retinoid transactivation of two hepatic transporter genes involved in bile formation J Biol Chem 2000 275 835 8843 10.1074/jbc.275.12.8835 10722729

[B22] Denson LA Bohan A Held MA Boyer JL Organ-specific alterations in RAR alpha: RXR alpha abundance regulate rat Mrp2 (Abcc2) expression in obstructive cholestasis Gastroenterology 2002 123 599 607 10.1053/gast.2002.34758 12145812

[B23] Roelofsen H Schoemaker B Bakker C Ottenhoff R Jansen PL Elferink RP Impaired hepatocanalicular organic anion transport in endotoxemic rats Am J Physiol 1995 269 G427 G434 7573454 10.1152/ajpgi.1995.269.3.G427

[B24] Whiting JF Green RM Rosenbluth AB Gollan JL Tumor necrosis factor-alpha decreases hepatocyte bile salt uptake and mediates endotoxin-induced cholestasis Hepatology 1995 22 1273 1278 7557881 10.1016/0270-9139(95)90639-8

[B25] Trauner M Arrese M Soroka CJ Ananthanarayanan M Koeppel TA Schlosser SF Suchy FJ Keppler D Boyer JL The rat canalicular conjugate export pump (Mrp2) is down-regulated in intrahepatic and obstructive cholestasis Gastroenterology 1997 113 255 264 10.1016/S0016-5085(97)70103-3 9207286

[B26] Vos TA Hooiveld GJ Koning H Childs S Meijer DK Moshage H Jansen PL Müller M Up-regulation of the multidrug resistance genes, Mrp1 and Mdr1b, and down-regulation of the organic anion transporter, Mrp2, and the bile salt transporter, Spgp, in endotoxemic rat liver Hepatology 1998 28 1637 1644 10.1002/hep.510280625 9828229

[B27] Geier A Dietrich CG Voigt S Kim SK Gerloff T Kullak-Ublick GA Lorenzen J Matern S Gartung C Effects of proinflammatory cytokines on rat organic anion transporters during toxic liver injury and cholestasis Hepatology 2003 38 345 354 12883478 10.1053/jhep.2003.50317

[B28] Chen HL Liu YJ Chen HL Wu SH Ni YH Ho MC Lai HS Hsu WM Hsu HY Tseng HC Jeng YM Chang MH Expression of hepatocyte transporters and nuclear receptors in children with early and late-stage biliary atresia Pediatr Res 2008 63 667 673 10.1203/PDR.0b013e318170a6b5 18327154

[B29] Davenport M Gonde C Redkar R Koukoulis G Tredger M Mieli-Vergani G Portmann B Howard ER Immunohistochemistry of the liver and biliary tree in extrahepatic biliary atresia J Pediatr Surg 2001 36 1017 1025 10.1053/jpsu.2001.24730 11431768

[B30] Mack CL Falta MT Sullivan AK Karrer F Sokol RJ Freed BM Fontenot AP Oligoclonal expansions of CD4+ and CD8+ T-cells in the target organ of patients with biliary atresia Gastroenterology 2007 133 278 287 10.1053/j.gastro.2007.04.032 17631149 PMC1949019

[B31] Nuclear Receptors Nomenclature Committee A unified nomenclature system for the nuclear receptor superfamily Cell 1999 97 161 163 10219237 10.1016/s0092-8674(00)80726-6

[B32] Kast HR Goodwin B Tarr PT Jones SA Anisfeld AM Stoltz CM Tontonoz P Kliewer S Willson TM Edwards PA Regulation of multidrug resistance-associated protein 2 (ABCC2) by the nuclear receptors pregnane × receptor, farnesoid Xactivated receptor, and constitutive androstane receptor J Biol Chem 2002 277 2908 2915 10.1074/jbc.M109326200 11706036

[B33] Zollner G Trauner M Nuclear receptors as therapeutic targets in cholestatic liver diseases Br J Pharmacol 2009 156 7 27 10.1111/j.1476-5381.2008.00030.x 19133988 PMC2697779

[B34] Paumgartner G Beuers U Ursodeoxycholic Acid in Cholestatic Liver Disease: Mechanisms of Action and Therapeutic Use Revisited Hepatology 2002 36 525 531 10.1053/jhep.2002.36088 12198643

